# Adoptive cell therapy for high grade gliomas using simultaneous temozolomide and intracranial mgmt-modified γδ t cells following standard post-resection chemotherapy and radiotherapy: current strategy and future directions

**DOI:** 10.3389/fimmu.2024.1299044

**Published:** 2024-02-07

**Authors:** L. B. Nabors, L. S. Lamb, T. Goswami, K. Rochlin, S. L. Youngblood

**Affiliations:** ^1^ Department of Neurology, University of Alabama at Birmingham, Birmingham, AL, United States; ^2^ IN8Bio, Inc., New York, NY, United States

**Keywords:** glioblastoma, genetic engineering, DNA damage (DDR), T cells gamma delta, cell therapy

## Abstract

Cellular therapies, including chimeric antigen receptor T cell therapies (CAR-T), while generally successful in hematologic malignancies, face substantial challenges against solid tumors such as glioblastoma (GBM) due to rapid growth, antigen heterogeneity, and inadequate depth of response to cytoreductive and immune therapies, We have previously shown that GBM constitutively express stress associated NKG2D ligands (NKG2DL) recognized by gamma delta (γδ) T cells, a minor lymphocyte subset that innately recognize target molecules via the γδ T cell receptor (TCR), NKG2D, and multiple other mechanisms. Given that NKG2DL expression is often insufficient on GBM cells to elicit a meaningful response to γδ T cell immunotherapy, we then demonstrated that NKG2DL expression can be transiently upregulated by activation of the DNA damage response (DDR) pathway using alkylating agents such as Temozolomide (TMZ). TMZ, however, is also toxic to γδ T cells. Using a p140K/MGMT lentivector, which confers resistance to TMZ by expression of O(6)-methylguanine-DNA-methyltransferase (MGMT), we genetically engineered γδ T cells that maintain full effector function in the presence of therapeutic doses of TMZ. We then validated a therapeutic system that we termed Drug Resistance Immunotherapy (DRI) that combines a standard regimen of TMZ concomitantly with simultaneous intracranial infusion of TMZ-resistant γδ T cells in a first-in-human Phase I clinical trial (NCT04165941). This manuscript will discuss DRI as a rational therapeutic approach to newly diagnosed GBM and the importance of repeated administration of DRI in combination with the standard-of-care Stupp regimen in patients with stable minimal residual disease.

Introduction

Newly diagnosed GBM, like many cancers, is first treated with a combination of surgery, induction radiation and chemotherapy followed by maintenance chemotherapy and subsequently monitored for recurrent disease, which is almost without exception a certainty. Once the recurrent tumor is evident and, depending on tumor characteristics and patient eligibility, clinical trials become available. Unfortunately, once the recurrent tumor becomes visible to imaging protocols it is already well past our ability to prevent the eventual uncontrolled proliferation and ultimately death. Upon recurrence, despite single or multi-agent chemotherapy, or surgery, nothing has shown an overall survival benefit and the median survival is approximately 8 months. Therefore, generating deeper tumor responses and delaying the time to tumor regrowth at first diagnosis are the best means to improve overall survival and quality of life for patients. We present a novel approach in which we seek to recapitulate the natural immunosurveillance function of innate recognition and control of GBM with primary standard-of-care therapy to create advantages for immune recognition and persistent surveillance.

## The immune system and glioblastoma

The concept of cancer immunosurveillance predicts that the immune system can recognize precursors of cancer and, in most cases, destroy these precursors before they become clinically apparent. Animals that possess naturally occurring or experimentally induced defects leading to loss of recombination-activating gene 2 (RAG2), αβ T cells, γδ T cells, invariant NKT cells, interferon-γ (IFNγ) receptor 1 (1); signal transducer and activator of transcription 1 (STAT1), perforin; or tumor-necrosis factor (TNF)-related apoptosis-inducing ligand (TRAIL) (2) are more susceptible to spontaneous development of cancer or carcinogenic stimuli. Accordingly, the immune system is known to generate a coordinated response against pre-malignant cell clusters and developing tumors. For instance, the DDR evident in GBM and several other cancers can induce expression of tumor-associated stress receptors including NKG2D ligands (NKG2DL) such as MHC-class-I-polypeptide-related sequence A (MICA) and UL-16 binding proteins (ULBP) 1-8 thereby sensitizing malignant cells to killing by the immune system’s NKG2D receptor-expressing first responders, such as NK cells, NKT cells, γδ T cells and some CD8^+^ αβ T cells. T cell-mediated adaptive immune responses are also induced in concert with this broad-based stress-associated response as tumor-associated antigens (TAA) are presented to T cells via MHC class I or II on antigen presenting cells (APC) which then trigger T cell activation and expression of co-stimulatory molecules and secretion of chemokines and cytokines. Clonal expansion of TAA-specific T cells then occurs as well as other immune effector cells that regulate different aspects of the immune response. Direct cell-mediated cytotoxicity as well as an indirect antibody complement-mediated cytotoxicity (3) are both employed in the adaptive response. Despite heightened immune function in the premalignant stage, tumor cells can escape and disseminate. In particular, GBM exists in an environment that is generally protected from a robust immune response given the relatively immune privileged nature of the brain when compared to other systemic cancers and can grow undetected until its mass is of sufficient size to provoke symptomatic neurologic dysfunction.

The core standard of care for primary GBM was defined in 2005 by Stupp (4) and remains to date, the most widely used treatment regimen. With some variation, the Stupp regimen begins with gross total resection, the extent of which is dependent on retaining function of nearby areas of the brain that execute critical sensory and/or motor functions. Following resection, the patient recovers for 3-4 weeks and then receives a 6-week therapeutic combination of targeted radiation and daily TMZ, followed by six 28-day maintenance cycles consisting of five consecutive days of oral TMZ at the initiation of each cycle. The median survival from diagnosis for patients receiving this regimen is 15 months although this figure is variable and largely dependent on the genotypic characteristics of the tumor. Despite the gains achieved by primary debulking, radiation therapy, and maintenance, this regimen unfortunately enables the selection of resistant genomic variants that will eventually outlast every therapy presently available for recurrent disease.

Since the immune system is known to respond to and combat tumors including GBM, it would seem logical that adjunct immunotherapy regimens might be effective in reducing tumor burden and improving progression free survival (PFS). Preclinical models have suggested effectiveness, however, GBM has been remarkably resistant to immunotherapy protocols including checkpoint inhibition and CAR-T therapies that have been advanced to the clinic. This may be partially due to the natural interaction between the brain and the immune system which is inherently biased against destructive inflammatory responses. The GBM tumor microenvironment (TME) contains a large proportion of immunosuppressive myeloid cells that can attenuate the T cell responses required for effective anti-tumor responses. Accordingly, immune checkpoint blockade has shown little efficacy in the adjuvant setting (5), although the neoadjuvant setting has shown some promise (6). Despite the remarkable outcomes seen with hematologic malignancies, immune cell therapies such as chimeric antigen receptor (CAR) T cell therapies have been generally disappointing in solid tumors to-date. CAR-T programs targeting the interleukin-13 receptor (IL13R)α2 (7), epidermal growth factor receptor variant III (EGFRvIII) (8) and other potential targets have been generally well-tolerated and have produced extended stable disease and/or long-term remission in some patients with recurrent GBM. However, the biologic characteristics of GBM discussed above including the immunosuppressive tumor microenvironment, tumor-derived systemic immunosuppression, antigenic heterogeneity, on-target off-tumor toxicities, and T cell exhaustion have been formidable barriers to successful immunotherapy of GBM.

## γδ T cells and the recognition of malignant disease – multiple weapons, multiple targets

γδ T cells are thought to be multi-specific, and antigen recognition demonstrates remarkable diversity (9). These T cells can recognize malignant cells through less specific mechanisms that do not require prior antigen exposure or priming, a function that is shared by other innate immune cells such as macrophages and NK cells. Unfortunately, the tumor responses of adoptive cellular therapies against hematopoietic cancers have not, with rare exceptions, been replicated in solid tumors such as GBM. The immunogenic heterogeneity of solid tumors even within a single tumor has frustrated attempts to target specific TAA (7, 10, 11) and has called for strategies that can more broadly distinguish and target malignant cells while still limiting the potential for damage to the host. More recently, Barish (12) showed that tumor antigen heterogeneity creates a significant challenge to tumor eradication. Their cohort of 44 high-grade brain tumor samples demonstrated four major histological regions of interest and significant antigen diversity within each individual region. Moreover, a CAR-T targeting three individual antigens, IL-13Rα2, EGFR and HER2 was still predicted to leave at least 7% of the tumor remaining (12). Additionally, Larson (13) demonstrated that loss of the interferon-γ receptor (IFNγR) signaling pathway rendered glioblastoma resistant to killing by CAR-T cells due to a reduction of the duration of cell binding and avidity. Consequently, the potential antineoplastic effect of γδ T cells, a minor T cell subset with distinct innate recognition properties, has recently become an area of intense investigation.

It is now known that γδ T cells play a critical role in tumor immunosurveillance (14–17) and in the immune response to cancer (18–23). In many instances, γδ T cells that are cytotoxic to a specific tumor type will cross-react with other tumors but not with the tumor’s non-transformed counterpart (21, 22, 24). Furthermore, the VγVδ2 subset of γδ T cells can respond early to infection or transformation and recruit adaptive responses from CD4+ and CD8+ T cells by internalizing antigens, processing them and displaying the antigens complexed with major histocompatibility complexes on their cell surface (25). As professional antigen presenting cells, γδ T cell lymphocytes express equivalent levels of costimulatory molecules and CCR7, home to lymph nodes and are equally potent at promoting proliferative responses in αβ T cells when compared to dendritic cells (9). Activating ligands for γδ T cells as well as the process by which they recognize stressed or malignant cells are complex and incompletely understood but are fundamentally different from both αβ T cells and NK cells (26–29).

The most prevalent circulating population of γδ T cells express an invariant Vγ9Vδ2 TCR (30). Vγ9/Vδ2+ T cells are thought to be activated via the T cell receptor (TCR) principally by three groups of non-peptide antigens: alkylphosphates such as isopentenyl pyrophosphate (IPP) generated by eukaryotic isoprenoid biosynthesis using the mevalonate pathway (31), alkylamines (32), and synthetic aminobisphosphonates (N-BP) (33, 34). Additionally, both Vδ1+ and Vδ2+ T cells express NKG2D, a C-type, lectin-like homodimeric activating receptor also expressed by NK cells and some αβCD8+ T cells. NKG2D is a ligand for MHC class-I like proteins such as major histocompatibility complex class I-related chain A/B (MICA/B), the UL-16 binding proteins (ULBP1-6) and MutS homologue 2 (MSH2). These NKG2D ligands provide a powerful danger signal to the immune system and are upregulated in response to cellular stress including infection and malignant transformation (35, 36). NKG2D ligation has been thought to play a costimulatory role in the activation of γδ T cells (37, 38), however, recent findings indicate that NKG2D ligation may be sufficient to independently activate certain γδ T cell subsets (39, 40). NKG2D activation is an important factor in tumor recognition and lysis by Vγ9Vδ2+ T cells, potentially playing a costimulatory role in cooperation with TCR-dependent activation (37, 41), although direct ligation of the Vγ9Vδ2+ receptor by the NKG2D ligand ULBP-4 has been reported (42). In some situations, NKG2D activation may be the primary stimulus, while TCR stimulation has a secondary role or is not required (40, 43).

## Resetting the clock - amplifying and extending the innate “first responder” paradigm

We have recently shown that ex vivo activated murine γδ T cells, when delivered intracranially during a period of minimal disease, failed to prevent tumor progression in a syngeneic GL261 mouse model (44) although they showed strong *in vitro* cytotoxic function against the same cell line. Prior to that study, we had also shown ex vivo human expanded and activated γδ T cells to be significantly effective in a human cell line xenograft model using a similar protocol (45). The apparent discordance was resolved by our observation that murine NKG2DL RAE-1 and MULT-1 are significantly downregulated in the hypoxic environment of the brain compared to that in the normoxic environment of ex vivo cell culture. Based on the observations of others who had shown that chemotherapy creates a favorable environment for a follow-on anti-tumor immune response, we then examined whether standard cytoreductive chemotherapy such as TMZ could increase stress antigen expression. Indeed, we were able to force transient upregulation of NKG2DL on chemotherapy resistant GBM cell lines with exposure to a therapeutic concentration of TMZ (46). The transient nature of this effect, however, precluded the timing of cell therapy administration outside of a pharmacokinetic point beyond which the cytotoxic effect on lymphocytes would also be at issue, particularly in a standard-of-care environment that would require five consecutive daily doses of TMZ. With that in mind, we generated a TMZ-resistant product by transducing γδ T cells with a p140K-MGMT expressing lentivector, a technique that had been previously used to build TMZ resistance into hematopoietic stem cells. TMZ-modified γδ T cells showed negligible losses and robust killing potential that was significantly improved in co-culture with GBM cell lines in TMZ-supplemented culture media (46). Finally, we tested the combination of intracranial therapy with MGMT-modified γδ T cells and TMZ against classical and mesenchymal primary and recurrent PDXT models in immunodeficient mice (47). Results showed significantly improved tumor-free survival at 150 days in mice with primary GBM PDXT receiving combination therapy over either single agent γδ T cells or TMZ for both classical and mesenchymal subtypes. Histopathology following sacrifice of survivors demonstrated an ability to target the heterogeneity of GBM tumors, with no discernable residual disease. Recurrent models fared poorly with a small effect of combination therapy noted in classical GBM subtype and no effect in mesenchymal PDXT. A separate safety study showed that the combination was not cytotoxic against cultured astrocytes exposed to radiation and/or TMZ chemotherapy and that NKG2DL were not upregulated on normal brain tissue from humans or mice exposed to stereotactic radiotherapy (48).

## Clinical trial design

**Figure 1:**
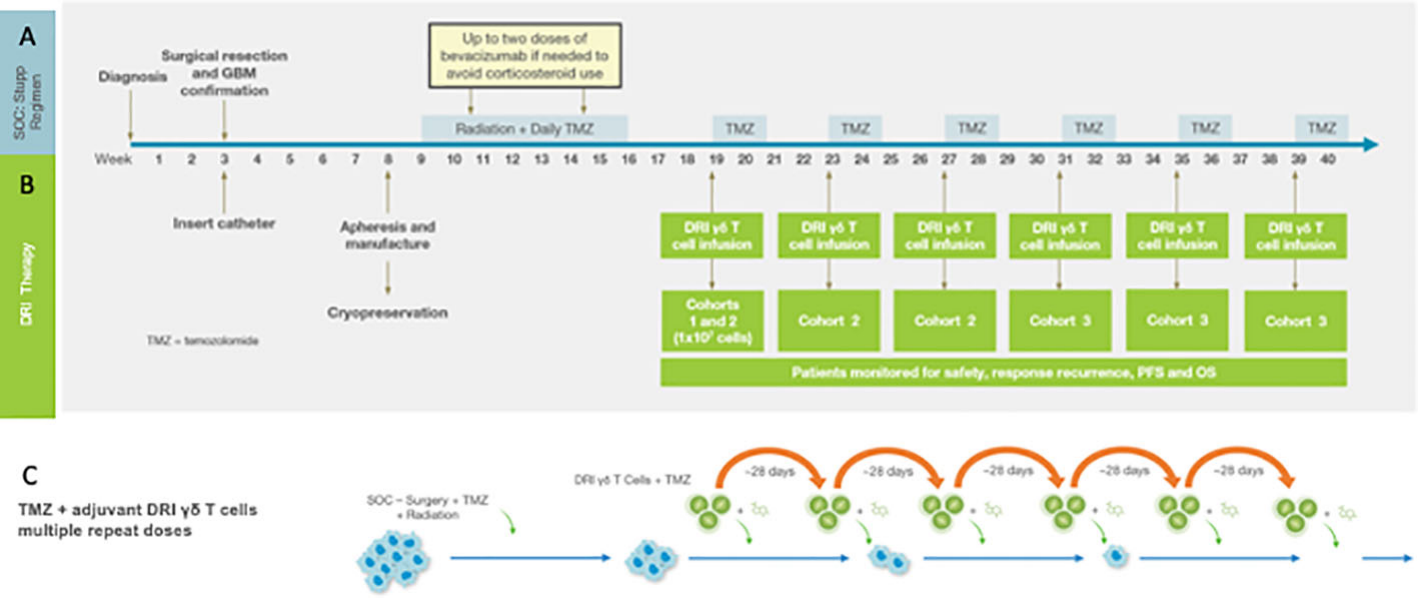
The Phase I Drug Resistant Immunotherapy trial is combined with the standard of care Stupp regimen consisting of resection + radiation/TMZ chemotherapy followed by six 28-day cycles of oral maintenance TMZ **(A)**. For the DRI protocol **(B)** a Rickham catheter is inserted into the tumor cavity following resection. Peripheral blood mononuclear cells (PBMC) for manufacturing of the cell product 3-4 weeks following tumor resection and prior to induction chemotherapy and radiotherapy (see text). The MGMT-modified γδ T cell product is infused on the first day of each maintenance cycle (depending on cohort-see text) within 4h of intravenous TMZ. **(C)** outlines the strategy that informs the clinical trial in which the tumor mass is reduced to minimal residual disease, the DDR and subsequent upregulated stress antigen expression then activated by TMZ and simultaneously targeted with a high local dose of MGMT-modified γδ T cells. Oral TMZ dosing continues for the remaining 4 days of the cycle and then repeated to provide continued pressure on tumor survival and proliferation.

These concepts – treatment of minimal residual primary tumor with innate γδ T cells following forced upregulation of tumor NKG2DL – are currently being explored in a Phase I clinical trial as a collaboration between the University of Alabama at Birmingham (UAB) and IN8Bio, Inc. The Stupp standard of care regimen is an ideal treatment platform to test the concept of repeated applications of high dose γδ T cell therapy in the setting of minimal residual disease. **Figure 1** details the Phase I trial design. Adult newly diagnosed GBM patients with adequate organ function and KPS>70% undergo gross total resection at which time a Rickham catheter (Integra LifeSciences; Princeton, NJ) is inserted into the resection cavity with a subcutaneous injection port placed under the skull. The patient then recovers for 3-4 weeks after which time an autologous mononuclear cell leukapheresis is obtained. Vγ9Vδ2 γδ T cells are expanded and activated using a proprietary manufacturing process (DeltEx™ DRI; IN8Bio, Inc., New York, NY) in media supplemented with Zoledronate (Novartis; Basel, Switzerland) and IL-2 (Miltenyi Biotech) in an automated bioreactor (Prodigy™: Miltenyi Biotec; Bergisch Gladbach, Germany) and transduced with the p140K-MGMT lentivector (Miltenyi Lentigen; Gaithersburg, MD). The final cell product is then harvested and cryopreserved in dose aliquots containing 1 x 10^7^ γδ T cells/cryovial.

It is well known that the circulating γδ T cell population is reduced in GBM patients by the dual-suppressing effects of exhaustion and tumor-derived systemic immunosuppression. Although zoledronate-mediated *in vivo* γδ T cell expansion has resulted in transient improvement for sensitive tumors (49), we have demonstrated that zoledronate + IL-2 mediated *in vivo* expansion of γδ T cells (50) results in only a moderate increase in the circulating γδ T cell count and expansion of the Treg population. Spacing resection and product collection provides time for recovery of cellular immunity as tumor-derived immunosuppressive cytokines decrease in the setting of minimal residual disease. Additionally, intracranial placement of the γδ T cells at the tumor site avoids the dilution and trapping of the cell product in the systemic microcirculation.

Dose administration begins on the first day of the first cycle of maintenance therapy where the patient receives the cell product through the intracranial Rickham catheter within 4 hours of intravenous (IV) TMZ. The remaining four TMZ doses are given orally, and the cycle repeats up to six times. In this dose escalation study, cohort 1 receives a single dose of γδ T cells on day 1 of Cohort 1 of maintenance while Cohort 2 receives γδ T cells on day one of cycles1-3 and Cohort 3 receives γδ T cells on cycles 1-6 along with temozolomide. In addition to standard of care diagnostic monitoring, patients are assessed at regular intervals for tumor genomics, histopathology, lymphocyte subsets, and serum cytokines. The primary endpoint is safety; secondary endpoints include progression free (PFS) and overall survival (OS). Dose limiting toxicities (DLTs) are defined as treatment related ≥ grade 3 cardiopulmonary or hepatic toxicity, grade 4 toxicity exceeding 72 hours or neurologic deterioration that exceeds 2 weeks.

This Phase I clinical trial (NCT04165941) is ongoing with anticipated completion of enrollment in 2023. Interim findings (51) for 15 enrolled patients (53% male; median age 69 (range: 21-76); 80% IDH-WT,66.7% MGMT unmethylated) of which 8 had been treated (N = 3 in C1, 4 in C2, 1 in C3) were presented at the 2023 American Society of Clinical Oncology conference.

## Biologic impediments, potential solutions, and future directions

Our design addresses several obstacles to effective prolonged tumor reduction that must be considered when developing γδ T cell-based cellular therapies (52). The first is that circulating γδ T cells from GBM patients are reduced in number and show impairment of proliferative function, thus limiting the applicability of autologous infusion therapies or strategies that rely solely on *in vivo* stimulation and expansion of γδ T cells (50). A separate though related problem is the sensitivity of normal γδ T cells to activation-induced cell death (AICD), which could impact the longevity of ex vivo expanded γδ T cells once infused (53). These issues have been anticipated and adopted into the manufacturing and therapeutic strategy. GBM-derived suppression of peripheral immunosuppression is known to decrease significantly following tumor resection (54), therefore the autologous cell product is obtained postoperatively and immediately prior to primary chemo/radiotherapy when immune recovery has occurred after tumor removal. Most importantly, given the heterogeneity of solid tumors, a more effective use of cell therapy may require a multi-pronged approach that relies on a more logical combinations and sequencing of each agent. Indeed, the rapid ability of tumors to expand requires rapid extraction and interruption of growth with surgery, chemotherapy and radiation and subsequent use of immunotherapy to eliminate residual tumor cells that may or may not be chemotherapy resistant. Once T cells have been successfully manufactured and infused, they can encounter an array of defensive measures that are generated by the tumor. Indeed, T cells must traverse the tumor vasculature (52), and survive tumor-derived inhibitory factors such as TGF-β and IL-10 which can inhibit antigen presentation, T cell activation, and expand of CD3+CD4+FoxP3+ regulatory T cells (55–57),, which have recently been implicated in the direct suppression of γδ T cell function (58). Tumor-derived proinflammatory factors also recruit monocyte-derived suppressor cells (MDSC) and mesenchymal stromal cells (MSC) into the tumor microenvironment (59) which can impair Vγ9Vδ2+ responses to phosphoantigen (60). Matrix metalloprotease derived proteolytic shedding of soluble NKG2D ligands can bind NKG2D (and possibly the γδ TCR) resulting in receptor endocytosis and inhibition of γδ T cell function (61). The repeated combination of TMZ chemotherapy and local application of MGMT modified γδ T cells over several months against small, undetectable malignant cell clusters should both reduce NKG2DL shedding, improve vulnerability to lysis by γδ T cells and inhibit formation of a vascularized and immunosuppressive tumor mass (62). Additionally, tumor-mediated effector-to-suppressor functional reprogramming of γδ T cells, which effectively results in a tumor-promoting γδ T cells phenotype, has been extensively documented for the Vδ1+ T cell population. Similar evidence for this effect for the Vγ9Vδ2+ population has not been documented in animal models or humans. Additionally, the cell therapy discussed herewith is intended for patients with minimal residual disease following subtotal resection and high-dose chemo/radiotherapy, which leaves the patient with no visible residual disease by standard imaging techniques, thereby lessening the potential effect of microenvironment that would be more characteristic of a bulky tumor. Finally, there is no evidence that expanded and activated Vδ2 T cell products are susceptible to reprogramming from effector to suppressor phenotype.

Additional combinations of chemotherapy, biologics, and CAR-T modifications to γδ T cells may further improve outcomes as these approaches move earlier in the treatment plan. Checkpoint inhibition, as shown earlier to be generally ineffective as a combination therapy with standard of care, presents an interesting biologic case if combined with γδ T cells. Tomogane (63) and Hoeres (64) recently examined the function of ex vivo expanded and activated γδ T cells across a variety of cell lines and found a decoupling between the anti-tumor cytotoxicity of γδ T cells and γδ T cell expression of PD-1 in that PD-1 blockade did not improve γδ T cell cytotoxicity against tumor lines. Interestingly, however, Tomogane showed a that a subset of PD-L1^high^ tumor lines were rendered more sensitive to ADCC-mediated γδ T cell lysis by PD-L1 blockade. Hoeres also showed that although PD-1 blockade did not improved cell-based cytotoxicity, it did upregulate IFN-γ production which could improve anti-tumor effect *in vitro*. We have previously shown (47) that PD-L1 is upregulated on GBM PDXT following treatment with TMZ which, as the review has noted, may impair DRI efficacy to some degree. Taken together, the probability exists that a neoadjuvant PD-1/PD-L1 regimen could improve overall γδ T cell function against a subset of PD-L1^high^ tumors although practical implementation would require further modeling.

Although we are hopeful that the strategy discussed above will lead to meaningful extension of PFS, we are cognizant of the unique challenges that GBM presents. The military principle of attacking a lightly defended position with overwhelming force and maintaining sustained pressure to prevent reinforcements (65) informs our strategy of repeated combination chemotherapy with a high dose of MGMT modified γδ T cells against a small population of residual primary tumor cells. With this approach we hope to minimize the immunosuppressive effect of the tumor, reduce the avenues for escape, and provide a path to sustained remission.

## Data availability statement

The original contributions presented in the study are included in the article/supplementary material. Further inquiries can be directed to the corresponding author.

## Ethics statement

The studies involving humans were approved by Western IRB. The studies were conducted in accordance with the local legislation and institutional requirements. The participants provided their written informed consent to participate in this study.

## Author contributions

LN: Investigation, Project administration, Resources, Writing – review & editing. TG: Project administration, Writing – review & editing, Methodology, Visualization. KR: Methodology, Project administration, Writing – review & editing, Investigation. SY: Investigation, Methodology, Project administration, Writing – review & editing, Resources. LL: Investigation, Project administration, Resources, Conceptualization, Funding acquisition, Supervision, Writing – original draft, Writing – review & editing.
